# Tele-Medicine Based and Self-Administered Interactive Exercise Program (Tele-Exergame) to Improve Cognition in Older Adults with Mild Cognitive Impairment or Dementia: A Feasibility, Acceptability, and Proof-of-Concept Study

**DOI:** 10.3390/ijerph192316361

**Published:** 2022-12-06

**Authors:** Catherine Park, Ram kinker Mishra, Michele K. York, Ana Enriquez, Abigail Lindsay, Gregory Barchard, Ashkan Vaziri, Bijan Najafi

**Affiliations:** 1 Interdisciplinary Consortium on Advanced Motion Performance (iCAMP), Michael E. DeBakey Department of Surgery, Baylor College of Medicine, Houston, TX 77030, USA; 2 VA’s Health Services Research and Development Service (HSR&D), Center for Innovations in Quality, Effectiveness, and Safety, Michael E. DeBakey VA Medical Center, Houston, TX 77030, USA; 3 Big Data Scientist Training Enhancement Program, VA Office of Research and Development, Washington, DC 20420, USA; 4 The BioSensics LLC, Newton, MA 02458, USA; 5 Neurology and Psychiatry & Behavioral Sciences, Baylor College of Medicine, Houston, TX 77030, USA

**Keywords:** dementia, exercise, telehealth, exergame, cognitive impairment, gamification, Alzheimer’s disease

## Abstract

Improved life expectancy is increasing the number of older adults who suffer from motor-cognitive decline. Unfortunately, conventional balance exercise programs are not tailored to patients with cognitive impairments, and exercise adherence is often poor due to unsupervised settings. This study describes the acceptability and feasibility of a sensor-based in-home interactive exercise system, called tele-Exergame, used by older adults with mild cognitive impairment (MCI) or dementia. Our tele-Exergame is specifically designed to improve balance and cognition during distractive conditioning while a telemedicine interface remotely supervises the exercise, and its exercises are gamified balance tasks with explicit augmented visual feedback. Fourteen adults with MCI or dementia (Age = 68.1 ± 5.4 years, 12 females) participated and completed exergame twice weekly for six weeks at their homes. Before and after 6 weeks, participants’ acceptance was assessed by Technology Acceptance Model (TAM) questionnaire, and participants’ cognition and anxiety level were evaluated by the Montreal Cognitive Assessment (MoCA) and Beck Anxiety Inventory (BAI), respectively. Results support acceptability, perceived benefits, and positive attitudes toward the use of the system. The findings of this study support the feasibility, acceptability, and potential benefit of tele-Exergame to preserve cognitive function among older adults with MCI and dementia.

## 1. Introduction

Motor and cognitive decline are common in older adults; they are closely associated and often interact because of shared brain networks [1,2]. By 2030, 20% of Americans (approximately 72 million people) will be 65 years or older [3], and by the age of 80 years or older, 40% or more will lose motor abilities [4]. In 2010, it was estimated that approximately 7% of older adults aged 65 years or older lived with cognitive impairment or dementia, and it was expected to double every 20 years [5]. Notably, multiple studies have reported that cognitive impairment is associated with an increased risk of falling, accelerating frailty development, a loss of independence in activities of daily living, increasingly higher healthcare costs, and poor quality of life [6,7,8,9,10].

Multiple systematic reviews have shown that various combinations of motor and cognitive training can improve motor-cognitive abilities in older adults for short (hours to days) and long (weeks to months) periods [11,12,13,14,15]. The most popular training protocol is physical-cognitive training, which produces new neuronal networks or strengthens synaptic activity [16]. For example, physical exercise training is performed simultaneously with a cognitive task in a dual-task manner (e.g., walking while counting backward from a random number, walking while performing a verbal fluency task, walking while reacting to signs and letters, etc.). Alternatively, separate cognitive tasks can be performed, followed by physical exercises. These physical-cognitive training programs have traditionally been delivered one-on-one or face-to-face in group settings in clinical and community facilities. However, cost, limited availability of physical therapists or health professionals, limited access to clinical and community facilities, and poor motivation hinder participation in physical-cognitive training regimens. Therefore, there have been growing attempts to develop technology-based physical-cognitive training regimens that facilitate accessibility, improve compliance and engagement, and enable user-tailored training [12,17].

Most technology-based motor-cognitive training systems use virtual reality (VR) or video games (exergames), which offer immersive and interactive training or intervention regimens (see Levin et al., 2017; Zhu et al., 2021 for review). These technology-based systems enable the providing of biofeedback (visual, auditory, or kinesthetic) or different levels of complexity of motor and cognitive tasks. Indeed, two systematic reviews have highlighted the beneficial effects of VR or exergame-based physical-cognitive training compared to conventional physical-cognitive training regimens in older adults with mild cognitive impairment (MCI) or dementia [12,17]. However, existing VR or exergame-based physical-cognitive training systems are unsuitable for telehealth training and monitoring due to the need for intensive resources (e.g., system complexity, time, cost), limited scalability, lack of remote feedback, and unavailability of monitoring training compliance and progress.

Recognizing these limitations and recent efforts to advance telehealth and telerehabilitation, we describe the design and development of a tele-Exergame system and assess the acceptance of interactive tele-Exergame by older adults with MCI or dementia. American Telemedicine Association defines tele-rehabilitation as delivering rehabilitation services via information and communication technologies (ICT) [18]. Based on this definition, the proposed tele-Exergame solution would promote tele-rehabilitation for older adults with cognitive decline.

This study adopts one of the most influential user acceptance and behavior analysis models, the Technology Acceptance Model (TAM), to analyze the factors influencing older adults with dementia’s acceptance of the tele-Exergame system. The TAM is a theoretical model that suggests that when a user is presented with a novel technology or application, a number of factors influence their behavioral intention to use the technology. The most influential factors are the perception of ease-of-use, and the usefulness of the technology perceived by the user. Recently, Mishra et al. (2022) used a TAM-based questionnaire to determine the acceptability of technology-driven solutions to facilitate dementia caregiving [19]. Similarly, several research studies used TAM-based questionnaires to determine the acceptability of novel technologies, including wearables, assistive devices, and web or mobile applications, among older adults with cognitive impairment [20,21,22].

We also describe cognition and anxiety levels in older adults with MCI or dementia after 6 weeks of in-home exergaming exercises. Previous studies assessed cognition and anxiety in combination with TAM constructs to identify any moderating effect of cognitive decline or high anxiety on responses to the TAM-based questionnaire. Key features of our tele-Exergame system include: (1) easy to use, (2) relatively inexpensive, (3) providing scalable physical-cognitive training, (4) storing user’s motor and cognitive performance, (5) providing feedback remotely, and (6) monitoring training compliance and progress remotely.

## 2. Materials and Methods

### 2.1. Tele-Exergame

**Figure 1:**
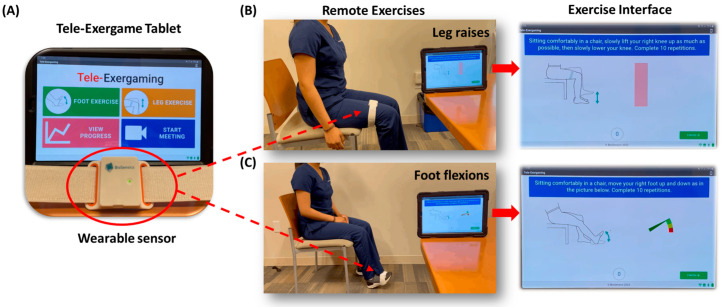
Tele-Exergame system. (**A**) Tablet and wearable sensor. (**B**) Leg raising exercise with the tele-Exergame system. (**C**) Foot flexion exercise with the tele-Exergame system.

Figure 1 shows an interactive tele-Exergame system, called Tele-FootX^TM^, that was designed and developed for people with MCI or dementia. Tele-FootX^TM^ consists of a motion sensor module (LEGSys^TM^, BioSensics, Newton, MA, USA) and a tablet (Galaxy Tab S5e, Samsung Electronics Co., Ltd., Suwon, South Korea) equipped with a custom application.

LEGSys^TM^ includes a triaxial accelerometer (±2 g) and gyroscope (±2000 deg/s), a microprocessor to process sensor data, and a Bluetooth module for wireless data acquisition. LEGSys^TM^ computes 3D rotation using the Kalman filter and quaternion approach, which is described in detail in our previous publications [23,24,25]. The sampling rate is 100 Hz, and 3D rotation data of LEGSys^TM^ are transmitted to the tablet via Bluetooth with high efficiency and zero package loss.

The custom application was developed using Java with Android Studio and with a user-centered approach by creating an intuitive user interface and minimizing the required manual interactions (Figure 1A). Functionalities of the custom application include: (1) pairing LEGSys^TM^ automatically when the sensor is detected, (2) administering two types of exercises (i.e., leg raising or foot flexion), (3) providing instructions using audio, images, and texts for the use of the tablet and LEGSys^TM^, (4) displaying user’s motion on the table’s screen by mapping the user’s leg or foot movements to dial’s displacements (Figure 1B,C) in real-time while performing exercises, (5) enabling remote monitoring of exercise progress and adherence, (6) providing remote coaching via an integrated video call, (7) storing exercise data on tablet’s internal memory, and (8) transferring saved exercise data to a cloud-based server for long-term storage and post-analyses without user’s private information.

### 2.2. Participants and Demographics

This study was registered at clinicaltrials.gov (Identifier: NCT05235113). Potential participants were recruited from the Baylor College of Medicine’s affiliated outpatient clinics or communities (Houston, TX, USA). Eligible participants were: (1) 65 years or older, (2) had clinically diagnosed MCI or dementia or had cognitive decline confirmed by the Montreal Cognitive Assessment (MoCA); the cutoff score on the MoCA was 25 or lower, (3) could walk at least 20 m with or without walking assistance, (4) were living independently in a residential home with a caregiver/informant, and (5) were willing and able to provide informed consent.

Exclusion criteria were as follows: (1) immobility or major mobility disorder or inability to engage safely in the proposed weight-bearing exercise program (e.g., double amputees, patients with active plantar ulcers, patients with major back or lower extremity pain); (2) severe cognitive impairment (MoCA score < 16); (3) other neurological conditions associated with cognitive impairment such as stroke, Parkinson disease, and head injury; (4) any clinically significant psychiatric condition, current drug or alcohol abuse, or laboratory abnormality that would interfere with the ability to participate in the study; and (5) major hearing/visual impairment. The study protocol was approved by the local Institutional Review Boards at Baylor College of Medicine (Protocols: H-44913). All participants read and signed a consent form prior to the study.

Demographics collected from all participants included age, gender, and body mass index (BMI). The following clinical information was also collected, such as high blood pressure, heart disease, musculoskeletal problems, stroke, depression, sleep problems, diabetes, osteoarthritis, rheumatoid arthritis, cancer, urinary tract problem, digestive problem, hearing problem, walking assistance use, fall history in the past year, daily prescription medications, and daily over-the-counter medications. Furthermore, all participants were assessed for their fear of falling using the Falls Efficacy Scale-International (FES-I) questionnaire [26,27] and their depression symptoms using the Center for Epidemiologic Studies-Depression (CES-D) questionnaire [27,28].

### 2.3. Experimental Procedures

The clinical psychologist (M.Y.) at Baylor College of Medicine confirmed the diagnosis of mild cognitive impairment or dementia. A trained research assistant interviewed the potential candidate for the study participation face to face. After confirming the satisfaction of the inclusion and exclusion criteria, informed consent was obtained, and the individual with dementia or MCI and their caregiver (if available) were trained with the tele-Exergame system. The clinical assessments, including administration of MoCA, BAI, CES-D, TAM questionnaire, and FES-I, were carried out in-clinic by the same research assistant.

At the beginning of 6 weeks of in-home exergaming exercises with the proposed tele-Exergame system, acceptance of tele-Exergame and participant’s cognition and anxiety were assessed (i.e., pre-assessment). To assess the acceptance of tele-Exergame by participants, the TAM questionnaire (Likert scale questionnaire), which is an intention-based model developed to evaluate user’s acceptance and user satisfaction with technology, was used [29]. The TAM questionnaire included eleven questions (two questions about user-friendliness and ease of use, seven questions about the perception of benefit, and two questions about the attitude towards use). Additionally, the MoCA was used to evaluate a participant’s cognition level [30], and the Beck Anxiety Inventory (BAI) questionnaire was used to evaluate a participant’s anxiety level [31].

All participants performed exergaming exercises (i.e., leg raising or foot flexion exercises) with the proposed tele-Exergame system twice per week for 6 consecutive weeks at their home. All participants were instructed to perform leg raising or foot flexion exercises for approximately 30 min every day. Participants were asked to wear the motion sensor (i.e., LEGSys^TM^) at the top of their foot for foot flexion exercises and the middle of their upper leg for leg raising exercises. After completing 6 weeks of in-home exergaming exercises, all participants were assessed for their acceptance of tele-Exergame, cognition, and anxiety by using the TAM questionnaire, MoCA, and BAI questionnaire (i.e., post-assessment).

### 2.4. Data and Statistical Analysis

Outcome measures included Likert scale points for each question of the TAM, MoCA score, and BAI score for two assessment periods (i.e., pre- and post-assessment). Statistical analysis was conducted using SPSS (IBM Corp., Armonk, NY, USA). A paired t-test was conducted to compare all outcome measures (i.e., Likert scale points for each question of the TAM, MoCA scores, and BAI scores) between two assessment periods (i.e., pre- and post-assessment). In addition, an effect size was calculated for all outcome measures using Cohen’s *d*. Values were defined as small (0.20–0.49), medium (0.50–0.79), large (0.80–1.29), and very large (above 1.30) [32]. Statistical significance was defined at the 2-sided *p* < 0.05 level.

## 3. Results

### 3.1. Demographic and Clinical Characteristics

Table 1 reports participants’ demographic and clinical characteristics, including fear of falling and depression. The average age and BMI were 68.1 ± 5.4 years and 31.5 ± 7.2 kg/m^2^, respectively. The average FES-I and CES-D scores were 25.7 ± 6.7 and 11.8 ± 10.7, respectively. A total of 35.7% and 28.6% of participants were identified as having a high concern of falling (i.e., FES-I score ≥ 28) and at risk for clinical depression (CES-D score ≥ 16).

### 3.2. Acceptance of Tele-Exergame

Table 2 reports the results of the TAM obtained before and after 6 weeks of in-home exergaming exercises with the proposed tele-Exergame system. The Likert scale rank included strongly disagree (4 points for Q1; 0 point for Q2–Q11), disagree (3 points for Q1; 1 point for Q2–Q11, neither disagree nor agree (2 points), agree (1 point for Q1; 3 points for Q2–Q11), or strongly agree (0 points for Q1; 4 points for Q2–Q11).

The results showed that participants agreed on user-friendliness and ease of use while performing in-home exergaming exercises with the proposed tele-Exergame system (i.e., Q1 and Q2). The Likert scale values were significantly decreased for the exergaming adherence (Q1) and increased for the participants’ level of interest while performing in-home exergaming exercises (Q2). The Likert scale values were significantly increased for Q3–Q9, which indicates that participants benefitted from performing in-home exergaming exercises to improve their physical and mental functions and quality of work. Specifically, participants felt significantly more energetic (Q3), improved self-awareness (Q4), increased mental alertness (Q5), improved quality of work (Q6), improved overall body functions (Q7), increased stamina (Q8), and improved flexibility (Q9) after 6 weeks of in-home exergaming exercises. Participants’ general attitude toward using the proposed tele-Exergame system was significantly increased. They indicated a willingness to perform similar or advanced exergaming exercises again (Q10), and they were satisfied with performing in-home exergaming exercises with the proposed tele-Exergame system (Q11). The Cohen’s d effect size was observed as large (0.80–1.29) for eight questions (i.e., Q1–2 and Q5–10) and very large (above 1.30) for three questions (i.e., Q3–Q4 and Q11).

Additionally, participants and their families also commented positively on the proposed tele-Exergame system and performing in-home exergaming exercises. For example, they mentioned that it was “Easy to learn how to use tablet and sensors. Believes it will improve her flexibility.”, “She likes that she is able to interact with modern technology because she does not have a computer at home”, “Exercises were the perfect intensity. Not too hard and not too easy.”, “She believes it will make her muscles more toned.”, “Believes that it improved his walking and ankle strength.”, “It gave me something to do every day. Motivated him to be more active and achieve the star rewards.”, etc.

### 3.3. Cognition and Anxiety

Table 3 reports the results of the MoCA and BAI obtained before and after 6 weeks of in-home exergaming exercises with the proposed tele-Exergame system. After completing 6 weeks of in-home exergaming exercises, participants significantly improved their cognition level and lowered their anxiety level. Specifically, the average MoCA score increased by 9.7%, and the average BAI score decreased by 27.6%. The Cohen’s d effect size was observed as small (0.20–0.49) for both MoCA and BAI scores.

## 4. Discussion

This proof-of-concept phase I clinical study aimed to design and develop an interactive tele-Exergame system, and quantitatively and qualitatively assess the acceptance of an interactive tele-Exergame by older adults with MCI or dementia. This study also quantitatively evaluated cognition and anxiety levels after completing 6 weeks of in-home exergaming exercises by older adults with MCI or dementia. The results showed that older adults with MCI or dementia experienced interactive exergaming exercises as interesting and motivating. Older adults with MCI or dementia indicated that they were able to use the proposed tele-Exergame system easily and considered it user-friendly. The exercises provided were inspired by the validated Buerger-Allen exercise program to prevent ankle deconditioning and improve walking capability [33]. Notably, their cognition and anxiety levels significantly improved after completing 6 weeks of in-home exergaming exercises.

The core innovation of the proposed system is the use of gamified safe balance tasks in an interactive environment, immediate and sensitive feedback about the user’s performance, and a motivating effect due to game-based features [34,35,36,37]. This is achieved by wearable sensors worn on the feet and the upper leg that measure lower extremity kinematics. The wearable sensors provide immediate external feedback about movement performance and motor errors, which may enhance motor learning and facilitate successful task execution. Studies suggest that focusing on movement results is more effective for motor learning than focusing on movement performance [38]. Augmenting movement results with external feedback can supplement internal feedback and serve as a ‘sixth sense’ [34]. This seems to be particularly important for patients with MCI and dementia, as well as those with impaired internal feedback, for instance, related to polyneuropathy, stroke, or Parkinson’s disease [34]. In addition, conventional tele-Exercise programs do not facilitate the quantitative remote assessment of cognitive-motor performance, which can assist with evaluating cognitive-motor improvement in response to exercise without the need to visit a clinic. The incorporation of wearable sensors into balance training has been repeatedly suggested in review articles [36,39]; however, to our knowledge, this approach has not yet been evaluated in patients suffering from MCI and dementia. Incorporating technology such as wearable sensors has been discussed as a promising option for improving balance and gait training regimes [34,36,40,41], and its effectiveness and advantages compared to traditional exercise programs have been discussed in a recent systematic review [37]. To our knowledge, however, this study is the first that implemented a tele-medicine-based, sensor-based and self-administered exercise program at home and demonstrated its feasibility and proof-of-concept effectiveness in people with MCI and dementia.

A major advantage of the proposed solution is its ability to improve access to care and health equity among older adults suffering from cognitive impairment. Many (40 to 80%) geriatric patients in rehabilitation are cognitively impaired [42,43]. Conventional balance programs in an unsupervised setting suffer from poor adherence [44], and may not be appropriate for cognitively impaired persons due to difficulties in correctly executing balance exercises [45]. Lack of motivation, health complaints, and transportation limitations have been identified as barriers to in-clinic exercise training in people with MCI or dementia [46]. Prior works [47,48] suggest that patients with worse baseline cognitive-motor performance may benefit most from exercise interventions, but at the same time, these individuals generally cannot regularly visit a rehabilitation center for face-to-face exercise, have the highest drop-out rates because of functional limitations, and have the lowest rate of adherence to unsupervised exercise programs [46,47,49]. Therefore, a tele-exercise program tailored for patients with cognitive impairment is needed. Such a system could help to preserve cognitive function in people with MCI or dementia, promote independent living, ensure patients in rural areas do not face undue hardships associated with receiving rehabilitation, and reduce costs associated with various consequences of cognitive-motor decline, including falls and frailty. The proposed tele-Exergame solution may address these gaps by offering virtual coaching to support the accurate execution of exercises while adding cognitive components. Additionally, it enables remote tracking of the adherence to exercise and functional improvement via the tele-medicine interface offered by the tele-Exergame tablet.

A systematic review [50] on the use of tele-rehabilitation interventions on individuals with physical impairments showed that applying this methodology leads to clinical improvements that are generally equal to those induced by conventional face-to-face rehabilitation programs. In addition, a recent systematic review [51] provided evidence that cognitive tele-rehabilitation for neurodegenerative diseases may have comparable effects to conventional in-person cognitive rehabilitation, supporting the scientific premise for the effectiveness of such remote therapy for people with MCI. Although telerehabilitation was first documented in 1959, recent advances in telecommunications, mobile devices, wearable technologies, and the internet of things (IoT), have opened new horizons in telerehabilitation for different populations [52]. Telerehabilitation’s main benefits include delivering prolonged therapeutic regimens tailored to patients’ needs remotely, cost- and time-effectively, strengthening the patient-provider connection, and improving health care, particularly for patients with geographic or mobility limitations. Indeed, telerehabilitation can be suitable for older adults with MCI or dementia, because most older adults diagnosed with MCI or dementia are under care at home and require longitudinal and continuous care [53].

Recent clinical studies and meta-analyses indicate that technology-based motor-cognitive training systems are feasible and can improve motor-cognitive functions in older adults with MCI or dementia, comparable to conventional clinical motor-cognitive training. For example, a systematic review and meta-analysis found that computerized cognitive training incorporating a VR system or using videogames provided by Nintendo Wii improved global cognition, attention, and working memory in older adults with MCI or dementia [54]. Another systematic review and meta-analysis reported that older adults with MCI or dementia improved their motor-cognitive functions after completing dual-task exergame interventions using the Xbox Kinect system [14]. The findings of these systematic reviews and meta-analyses are consistent with our results that older participants with MCI or dementia improved their cognitive level after completing 6 weeks of in-home exergaming exercises with our interactive tele-Exergame system. Our results showed that older participants with MCI or dementia lowered their anxiety level, as supported by a decrease in anxiety levels in older adults after conventional cognitive training [55].

Our tele-Exergame system has several advantages over existing VR- or videogame-based systems or exergame systems using Kinect. It uses a relatively inexpensive motion sensor to measure user’s motion in real time and support motor-cognitive exercises. Notably, our tele-Exergame system enables the remote monitoring of exercise progress and adherence, and remote coaching and consultation via an integrated video call. This functionality could potentially contribute to enhancing the health care providers’ knowledge about the patients by facilitating patient education and establishing shared goal setting and action planning for rehabilitation interventions. Furthermore, our custom application can be implemented on smartphones that are already universal and ubiquitous all around the world. In 2011, the smartphone’s penetration rate is approximately 97% in the United States and continues to increase worldwide [56,57]. People aged 50 to 64 years and aged over 65 years own smartphones at the rate of 83% and 61%, respectively [56,57]. Therefore, our system’s capability to support smartphones can facilitate use of telerehabilitation to improve motor-cognitive functions in older adults. Indeed, smartphone-based technology has several benefits in terms of cost, size, weight, portability, accessibility, telecommunication, and in the ability to connect various external sensors/devices (e.g., motion sensor, physical activity monitoring sensor, heart rate monitor, etc.) [58].

The limitations of the present study are a relatively small sample size, and the lack of a control group (i.e., older adults with MCI or dementia receiving typical paper-based regimens). An additional limitation is a relatively short period of in-home exergaming exercises (i.e., 6 weeks). A recent systematic review and meta-analysis reported that the number of interventions ranged from 1 to 3 days per week, and the longest exercise duration was 16 weeks [14]. Despite these limitations, this proof-of-concept study showed that our tele-Exergame system effectively motivates and encourages older adults with MCI or dementia to perform in-home exergaming exercises, and improves cognition and anxiety levels after older adults with MCI or dementia complete 6 weeks of in-home exergaming exercises.

## 5. Conclusions

This study is the first step toward implementing a tele-medicine based and interactive home-based exercise program tailored for people with MCI or dementia. The proposed exergaming technology supports cognitive-motor exercises that have been designed to improve balance and cognition during distractive conditioning. It also enables remote monitoring of exercise progress and adherence as well as remote coaching and consultation. The results showed the feasibility and acceptability of home-based and self-administered interventions for people with MCI and dementia. While the results are preliminary, they support the potential effectiveness in engaging older adults with MCI and dementia in regular cognitively demanding exercise tasks at home, which potentially helps improve or preserve cognitive function and reduce anxiety. These encouraging results pave the way for future randomized control trials (RCT) to demonstrate the effectiveness of encouraging this home-based exercise program to improve cognitive and motor functions in older adults with MCI and dementia.

This study was a phase I of a larger randomized control trial described in clinicaltrials.gov (Identifier: NCT05235113). In the next phase study (Phase II), we plan to examine the effectiveness of a 12-week tele-Exergame program using a RCT design, with additional primary and secondary outcomes including motor performance (gait and balance), mobility (e.g., daily steps), cognition, patient reported outcomes (e.g., depression, quality of life, sleep quality, fear of falling), and risk of falling. The eventual goal is to provide a self-administered, remotely supervised home-exercise program for older adults with MCI or dementia to help them preserve cognitive-motor function while reducing the consequences of cognitive-motor decline such as reduced mobility, falls, anxiety, depression, and reduced independency.

## Figures and Tables

**Table 1:** Demographic and clinical characteristics, including fear of falling and depression at the baseline.

	Mean ± Standard Deviation or N (%)
Number of participants	14
Demographics	
Age, years	68.1 ± 5.4
Gender (Female)	12 (85.7)
BMI, kg/m^2^	31.5 ± 7.2
Clinical characteristics	
High blood pressure	12 (85.7)
Heart disease or circulation problems	1 (7.1)
Musculoskeletal problems	5 (35.7)
Stroke	1 (7.1)
Depression	5 (35.7)
Sleep problems, n	7 (50.0)
Diabetes	5 (35.7)
Osteoarthritis	2 (14.3)
Rheumatoid arthritis	2 (14.3)
Cancer	1 (7.1)
Urinary tract problems	2 (14.3)
Digestive problems	5 (35.7)
Hearing problems	3 (21.4)
Walking assistance use	4 (28.6)
Had fallen in the past year	1 (7.1)
Daily prescription medications, number	5.4 ± 2.5
Daily over-the-counter medications, number	0.9 ± 1.3
Fear of falling and Depression	
Concern for fall (FES-I score)	25.7 ± 6.7
High concern, FES-I ≥ 28	5 (35.7)
Depression (CES-D score)	11.8 ± 10.7
Depressed, CES-D ≥ 16	4 (28.6)

Values are presented as mean ± standard deviation or n (%). BMI: Body Mass Index; FES-I: Falls Efficacy Scale-International; CES-D: Center for Epidemiologic Studies-Depression.

**Table 2:** Statistical analysis results of the Technology Acceptance Model (TAM) questionnaire.

Question		Pre	Post	Mean Difference	Cohen’s *d*	*p* Value
User-friendliness and ease of use						
Q1	I tried very hard during this exercise.	2.0 ± 1.4	0.9 ± 1.2	−55.0%	0.84	0.032 *
Q2	I would describe this training exercise as very interesting.	3.1 ± 0.3	3.5 ± 0.5	12.9%	0.97	0.037 *
Perception of Benefit						
Q3	I feel more energetic at home after doing exercise.	3.0 ± 0.5	3.7 ± 0.5	23.3%	1.40	0.025 *
Q4	Exercise improves my self-awareness.	3.1 ± 0.3	3.7 ± 0.5	19.4%	1.46	0.005 *
Q5	Exercising increases my mental alertness.	2.9 ± 0.7	3.5 ± 0.7	20.7%	0.86	0.005 *
Q6	Exercise improves the quality of my work.	3.1 ± 0.6	3.7 ± 0.5	19.4%	1.09	0.024 *
Q7	Exercise improves overall body functioning for me.	3.2 ± 0.4	3.7 ± 0.5	15.6%	1.10	0.015 *
Q8	Exercise increases my stamina.	3.2 ± 0.4	3.7 ± 0.5	15.6%	1.10	0.015 *
Q9	Exercise improves my flexibility.	3.2 ± 0.4	3.7 ± 0.5	15.6%	1.10	0.015 *
Attitude towards Use						
Q10	I will do similar or more advanced tele-Exergame again.	3.2 ± 0.4	3.6 ± 0.5	15.6%	1.10	0.037 *
Q11	Overall, I am very satisfied with the tele-Exergame program I received.	3.1 ± 0.6	3.8 ± 0.4	22.6%	1.37	0.010 *

Values are presented as mean ± standard deviation or n (%). Asterisk denotes a significant difference between the assessment period.

**Table 3:** Statistical analysis results of the Montreal Cognitive Assessment (MoCA) and Beck Anxiety Inventory (BAI).

	Pre	Post	Mean Difference	Cohen’s *d*	*p* Value
Montreal Cognitive Assessment (MoCA) score	20.6 ± 5.0	22.6 ± 5.6	9.7%	0.38	0.017 *
Beck anxiety inventory (BAI) score	9.6 ± 8.4	6.9 ± 10.1	−27.6%	0.29	0.019 *

Values are presented as mean ± standard deviation or n (%). Asterisk denotes a significant difference between the assessment period.

## Data Availability

The de-identified datasets (except sensor data) are available upon request to the corresponding author.
